# Surface and vertical dimensional changes of mineral trioxide aggregate and biodentine in different environmental conditions

**DOI:** 10.1590/1678-7757-2018-0093

**Published:** 2018-12-10

**Authors:** Hacer Aksel, Selen Küçükkaya Eren, Sevinc Askerbeyli Õrs, Eda Karaismailoğlu

**Affiliations:** 1 Hacettepe University, Faculty of Dentistry, Department of Endodontics, Ankara, Turkey; 2 Kastamonu University, Faculty of Medicine, Department of Biostatistics, Kastamonu, Turkey

**Keywords:** Biodentine, Calcium silicate-based cement, MTA, Profilometer, Roughness

## Abstract

Surface changes in biological environments are critical for the evaluation of physical and biological activity of biomaterials. Objective: This study investigated surface alterations of calcium silicate-based cements after exposure to different environments. Material and Methods: Forty-eight cylindrical cavities were prepared on root surfaces. The cavities were filled using ProRoot MTA or Biodentine and assigned to four subgroups (n=6): dry, wet, acidic, and blood. Surface topographies were evaluated using an optical profilometer for 28 days, and the roughness of the material surfaces was quantified. Vertical dimensional change was measured by determining the height difference between the material surface and the flat tooth surface. Data were compared with a two-way repeated measures ANOVA and Bonferroni tests. Results: In dry condition, the surface roughness of MTA or Biodentine was constant up to 3 days (p>0.05) but decreased after 28 days (p<0.05). In dry condition, ProRoot MTA presented constant surface level through time, while Biodentine showed decreased surface level after 28 days. In wet condition, the roughness and the surface levels of both materials increased after 1 day (p<0.05). Neither the surface roughness nor the levels of the materials showed significant changes in acidic conditions (p>0.05). Both materials showed the highest roughness in blood conditions on the 1^st^ day (p<0.05), while the surface roughness in blood decreased dramatically after 28 days. The roughness of Biodentine was higher in wet conditions up to 3 days compared with ProRoot MTA (p<0.05). Likewise, in blood condition, Biodentine showed higher roughness on the 28^th^ day than ProRoot MTA (p<0.05). Conclusions: Dry, wet, and blood conditions had a time-dependent effect on the surface roughness and vertical dimensional changes of the materials. However, acidic conditions did not affect the roughness and the surface level of the materials.

## Introduction

Mineral trioxide aggregate (MTA) and Biodentine are mostly used calcium silicate-based cements (CSCs) in several endodontic procedures, such as retrograde filling, coronal barrier, pulp capping agent, and perforation repair material. ^1^ Both materials consist of tricalcium silicate and dicalcium silicate as main components. MTA also contains calcium sulfate and bismuth oxide (radiopacifier), and Biodentine contains calcium carbonate and zirconium oxide (radiopacifier). Hydration of the materials forms calcium silicate hydrate gel and calcium hydroxide. ^2^ MTA completes the initial setting after 40 min and solidifies completely in 140 min. ^3^ Biodentine has a smaller particle size that increases the surface area and density of the material compared with MTA. ^4^ Biodentine has a relatively short setting time and showed initial setting after 9-12 min and solidifies after 45 min. ^5^

The local physicochemical environment determines the suitability of the materials for clinical applications. ^6^ MTA and Biodentine generally come into contact with body fluids and moisture when used for endodontic applications, and both materials can solidify in blood, plasma, and other fluids. ^7^ However, when these materials are used as cavity liners and bases under final adhesive restorations, they are placed in relatively dry conditions. ^8^ In addition, the materials can be exposed to blood during endodontic applications, such as apical surgery and revascularization procedures. ^9^ The pH of the environment becomes acidic during endodontic treatment of necrotic teeth with periapical lesions or the repair of teeth with perforating furcal lesions, which may affect the properties of the materials. ^10^

Different oral conditions may affect the surface characteristics of the materials. ^11^ Surface roughness is a component of the surface texture and is also the measure of vertical (positive or negative) deviations of the surface from an ideal flat surface. The biocompatibility and bioactivity of these materials provide a microenvironment for cell attachment and odonto-/osteogenic activity during pulp capping procedures, ^12^ regenerative endodontics, ^13^ or periapical healing after apical plug formation. ^14^ According to previous studies, a rough surface may promote the attachment and proliferation of the cells by increasing material-cell interactions. ^15^ ^,^ ^16^ In addition, the materials can release calcium ions, and the accumulation of the calcium on the surface increases surface roughness, which leads to the formation of hydroxyapatite on the surface. ^17^ However, excessive surface roughness might have a negative impact on the strength and sealing of materials. ^18^ MTA and Biodentine have a similar surface roughness in wet conditions. ^15^ However, no comparable data on the effects of different conditions on the roughness of MTA or Biodentine were found in literature.

Setting conditions could also affect the dimensional stability of these materials. ^19^ The dimensional stability of a material should be adequate to improve its adaptation and prevent leakage. Slight expansion might contribute to the adaptation of the material, but excessive shrinkage or expansion during setting may lead to leakage, lack of marginal integrity, or cracks in the root canal walls. ^20^

Recently, an optical profilometer was used to measure the physical properties of dentin or the rotary instruments, as well as the adaptation of root end-filling materials. ^21^ The optical profilometer acquires a computer-based three-dimensional (3D) measurement of information on the surface characteristics of materials without damaging them during measurement. ^18^

This study attempted to reveal the surface characteristics of ProRoot MTA and Biodentine in different environmental conditions with a non-contact 3D optical profilometer.

## Material and Methods

The crowns of 24 human maxillary canine teeth were removed, and the root halves were obtained by sectioning the roots longitudinally. The middle part of each root half was used for the experiments. Each root half was embedded in a circular self-cure acrylic resin (Meliodent, Heraeus-Kulzer, Senden, Germany), and standardized cylindrical cavities (2 mm in height × 3 mm in diameter) were created in the middle part of each root half. A polishing device (Mecapol P230, Presi, Contamine-sur-Arve, France) using 600- and 1000-grit abrasive discs (Buehler, Lake Bluff, IL, USA) was used to polish the surface of the blocks. ^22^ Irrigation was performed during 3 min using 2.5% sodium hypochlorite (NaOCl), followed by 1 min of 17% ethylenediaminetetraacetic acid (EDTA) to remove any debris in the cavity.

The tooth halves were assigned to the MTA group or to the Biodentine group (n=24). To prepare the materials, 1 g of the powder of White ProRoot MTA (Dentsply Maillefer, Ballaigues, Switzerland) was mixed with 0.34 g liquid on a glass slab for 30 s, and Biodentine (Septodont, Saint Maur des Fausses, France) was prepared by pouring five liquid drops into the powder capsule and mixing for 30 s in a triturator. The materials were then inserted into the cavities using an MTA Endo gun (Dentsply Maillefer) and compacted with an endodontic plugger (Dentsply Maillefer) that was ultrasonically activated for 2 s using an ultrasonic tip CPR-1 (Dentsply Tulsa Dental, Tulsa, OK, USA) connected to a piezoelectric unit (Pmax, Satelec, Merignac, France) at the medium power setting. ^23^ The material surface was straightened from the surface of the specimens with a scalpel. The same operator prepared and placed the materials to prevent inter-operator discrepancies. After placement, the specimens in MTA or Biodentine groups were assigned to four subgroups according to the experimental condition (n=6): dry, wet (phosphate buffered saline: PBS; Biochrom, Berlin, Germany, pH 7.4), acidic (PBS, pH 5.0), or blood. To prepare the blood condition, human blood was obtained from a healthy volunteer, member of the research group, by phlebotomy using a 27-gauge needle. The blood was kept in a mold containing 50 IU of heparin (Nevparin, Mustafa Nevzat İlaç San. A.Ş., Istanbul, Turkey) *per* 1 mL of blood to prevent coagulation. The specimens were then incubated in 24-well plates with the respective environmental medium, and the wells were placed in an incubator with 100% humidity at 37°C for 28 days. The media were refreshed three times *per* week.

### Measurement of surface roughness

The surface of each specimen was scanned using a 3D optical profilometer (Zygo Corp., Middlefield, CT, USA). The image resolution was 640×480 pixels. The mean of the 3D surface roughness of each material was analyzed using image analysis software (MetroPro; Zygo Corp.). To do so, three images were taken of different parts of each material. The surface of each material was divided into three sections from left to right using analysis software to standardize the measurement area. The average roughness (Ra), root mean square (RMS), and maximum peak-to-valley height (PV) values were the roughness parameters used to determine the surface characteristics of the materials. ^24^ Ra represents the average roughness, RMS indicates the arithmetic mean of the height and depth of the surface from a mean line, and PV indicates the height distribution between the lowest and the highest peaks. The measurements for surface roughness analysis were performed for each sample at four different time points (45 min, 1, 3, and 28 days).

### Determination of vertical dimensional changes

**Figure 1:**
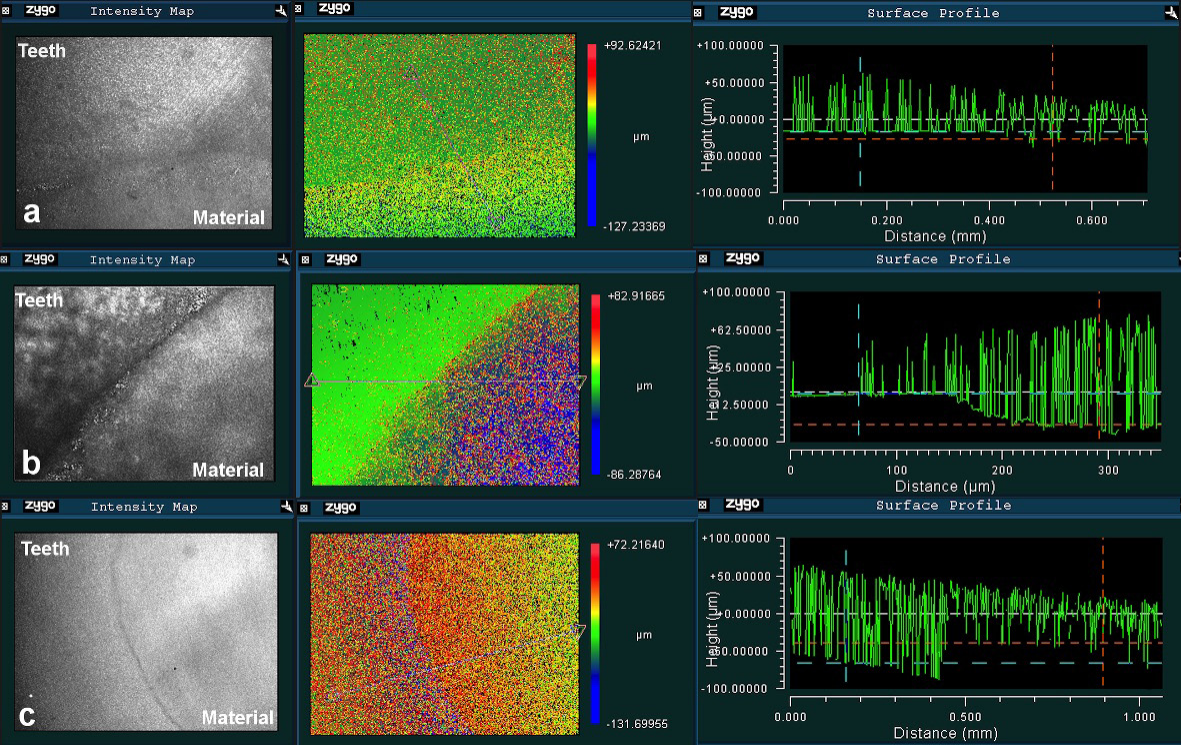
Representative 3D images for the measurement of height differences between the tooth surface and the material surface showing (a) constant, (b) decreased (shrinkage), and (c) increased (expansion) surface level of materials relative to the tooth surface

The vertical dimensional change (VDC) of the materials was measured by determining the height difference between the material surface and the flat tooth surface. VDC was determined by creating a straight plane from the tooth surface to the material surface. Three straight planes were created for each sample to measure different areas of the surface. The height difference was also determined by the visual assessment of the color differences; the color change from red to blue indicates a height change from the highest to the lowest surface ( Figure 1 ).

VDC measurements were performed for each sample at three different time points (up to 1, 3, and 28 days) using the surface level of each material at 45 min as the baseline.

### Statistical analysis

Changes in the surface roughness of ProRoot MTA and Biodentine in different conditions (dry, wet, acidic, and blood) at different time points (45 min, 1, 3 and 28 days) were compared using a two-way repeated measures analysis of variance (ANOVA) and Bonferroni tests. A p-value of <0.05 was considered significant.

The VDC changes in each condition were calculated as a percentage change from the baseline value.

## Results

### Surface roughness

**Figure 2:**
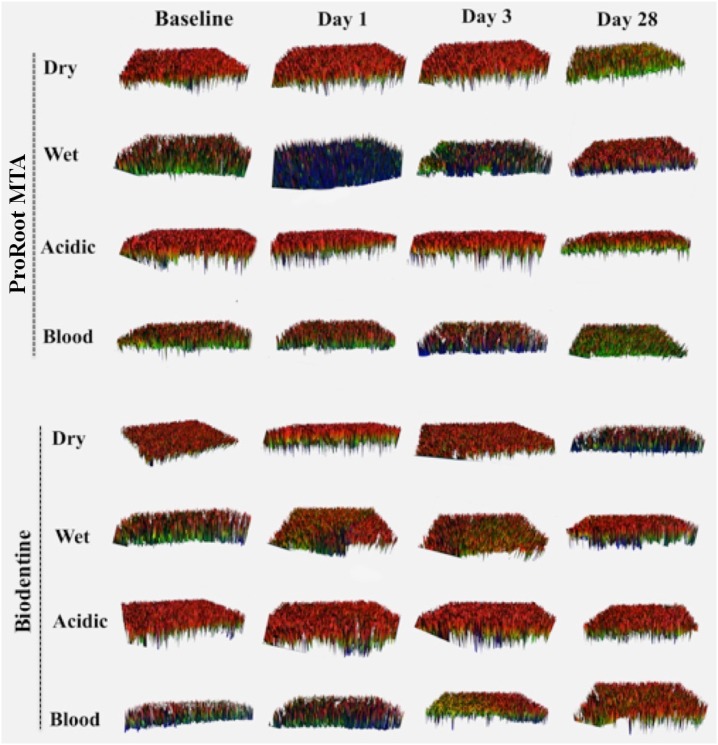
Representative 3D images obtained using an optical profilometer showing surface topographies of ProRoot MTA and Biodentine at different environmental conditions over time. The color change from red to blue indicates a surface change from increased to decreased roughness. 3D surface images show that the surface roughness of both materials decreased in dry conditions after 28 days. In wet conditions, the surface roughness of ProRoot MTA decreased on the 1^st^ day; however, roughness increased for up to 28 days for both materials. In acidic conditions, the surface roughness of both materials was similar at all time points. In blood conditions, the surface roughness of both materials decreased gradually after the 1^st^ day

Figure 2 shows the representative 3D surface topographies of the groups. Table 1 shows the mean ± SD of Ra, RMS, and PV values of each group. Statistical analysis revealed no significant differences in RMS and PV values among the groups (p>0.05). However, significant differences were found in Ra values depending on the experimental condition, storage time, and material (p<0.05).

**Table 1:** Time-dependent surface roughness values (mean ± SD) of ProRoot MTA and Biodentine in different environmental conditions

Parameter	Materials	Condition	45 min	Day 1	Day 3	Day 28
Avarage Roughness (Ra)	ProRoot MTA	Dry	17.09±2.95^a+^	15.09±3.42^a+^	16.41±3.72^a+^	9.88±2.19^b+^
		Wet	13.65±2.70^a+^	9.5±3.84^b*^	12.61±2.13^a,*^	23.64±1.85^c,*^
		Acidic	14.12±0.91 ^a+^	15.64±2.00^a,+^	15.50±1.50^a,+^	16.81±1.85^a,+^
		Blood	21.54±3.50^a,*^	25.21±2.85^b,#^	19.51±3.22^a,+^	**12.47±1.50^c,+^**
	Biodentine	Dry	15.74±3.66^a,+^	15.90±0.85^a,+^	15.06±1.26^a,+^	11.45±1.40^b,+^
		Wet	11.53±3.13^a,*^	**16.67±3.19^b,+^**	**17.51±2.88^b,+^**	23.35±5.74^c,^*
		Acidic	16.17±2.34^a,+^	14.02±0.84^a,+^	14.46±1.06^a,+^	16.62±3.43^a,#^
		Blood	21.94±4.20^a,#^	25.69±4.68^b,*^	22.88±5.80^b,*^	16.87±3.12^c,#^
Maximum peak-to-valley height (PV)	ProRoot MTA	Dry	106.78±2.21	111.79±11.56	104.73±1.58	119.9±9.3
		Wet	109.68±4.64	115.13±13.21	118.14±11.62	159.97±12.14
		Acidic	105.69±1.39	113.87±9.27	107.31±2.78	112.55±10.30
		Blood	166.68±3.92	163.53±6.56	172.94±11.18	119.43±14.43
	Biodentine	Dry	108.08±3.90	106.5±3.43	108.65±3.87	117.17±11.05
		Wet	114.81±3.99	128.32 ±7.12	119.63±16.23	161.75±13.30
		Acidic	113.22±12.91	107.71±3.10	103.89±0.99	120.04±17.14
		Blood	170.21±14.80	181.56±25.64	182.63±21.68	131.99±19.04
Root mean square (RMS) (root)	ProRoot MTA	Dry	21.55±3.03	18.94±3.75	20.67±4.01	14.11±1.95
		Wet	16.85±2.31	13.23±3.47	16.03±3.08	27.60±2.42
		Acidic	18.19±1.20	19.63±2.26	18.72±2.00	20.49±2.15
		Blood	25.99±3.85	29.50±2.92	24.45±3.27	16.51 ±1.42
	Biodentine	Dry	19.91±3.96	20.2±0.76	19.31±1.94	14.84±1.54
		Wet	15.48±2.95	20.62±3.47	21.33±3.18	29.29±5.25
		Acidic	20.31±2.36	18.08±1.11	18.65±1.27	20.86±3.48
		Blood	27.48±5.41	31.1±4.85	27.79±5.41	20.35±3.30

* In each row, different letters (a, b, c) indicate significant differences regarding storage time

* In each column, different symbols (+,*, #) show significant differences among the different test conditions

* Bold values show significant differences between the materials (p<0.05)

The surface roughness of ProRoot MTA and Biodentine in dry conditions was constant up to three days (p>0.05) but decreased markedly after 28 days (p<0.05). In wet conditions, the roughness of MTA decreased on the 1^st^ day, while the roughness of both materials increased after 1 day (p<0.05). In acidic conditions, both materials showed similar roughness, and the roughness values of each material did not change through time (p>0.05). In blood conditions, the surface roughness of both materials had the highest roughness values on the 1^st^ day, and they showed decreased roughness after 28 days (p<0.05).

The roughness of both materials was significantly higher in wet conditions compared with those after 28 days (p<0.05). After incubation period of 28 days, the roughness of ProRoot MTA was similar in blood and dry conditions (p>0.05), while Biodentine exhibited increased roughness in blood compared with dry conditions (p<0.05). Biodentine showed higher roughness compared with ProRoot MTA on days 1 and 3, after storage in wet conditions (p<0.05). The roughness of Biodentine was higher than ProRoot MTA in blood conditions on 28 days (p<0.05).

### Vertical dimensional changes

In dry conditions, the surface level of ProRoot MTA was similar at all time points, while the surface level of Biodentine showed 0.47% decrease after 28 days. In wet conditions, the surface levels of both materials remained constant up to the 1^st^ day. ProRoot MTA expanded 0.41% and 0.17%, while Biodentine expanded 0.85% and 1.44% on days 3 and 28, respectively. Both materials presented similar surface levels to the tooth surface in the acidic environment. The surface levels of both materials showed shrinkage in blood conditions. The decrease in the surface level of ProRoot MTA was 0.60%, 0.69%, 0.25%, while for Biodentine, it was 0.22%, 0.56% and 0.79% on days 1, 3 and 28, respectively.

## Discussion

In this study, a 3D optical profilometer was used to compare the effects of different environmental conditions on the surface roughness of ProRoot MTA and Biodentine over time. A 3D optical profilometer can record a wide range of surface changes ranging from <1 nm to 20000 μm in magnitude. In addition, it allows repeatable analyses at different time points without any sample preparation. It also allows the measurement of height variations relative to a known reference plane. The vertical dimensional change of materials was measured in previous studies using a dilatometer, a linear variable differential transducer. ^7^ ^,^ ^22^ ^,^ ^25^ This device contains a contact probe that records the dimensional change according to the probe position. However, the contact probe may limit the expansion of the material due to the pressure it exerts, and the sensitivity (±1 μm) might not be high to detect the small changes in the material. For this reason, a 3D optical profilometer was used to evaluate the vertical dimensional changes in ProRoot MTA and Biodentine in different conditions.

In clinical conditions, MTA and Biodentine are placed in direct contact with dentin. The interaction of MTA or Biodentine with dentin promotes a biomineralization process due to the dissolution of bioactive dentin matrix components and to the formation of carbonated apatite precipitates. ^26^ Dentin also has a high buffering capacity that can change the pH of the environment and the chemical and physical properties of the materials. ^27^ Therefore, the materials were placed into dentin cavities to simulate clinical conditions.

In this study, the surface roughness of ProRoot MTA and Biodentine in dry conditions was constant up to 3 days but decreased markedly after 28 days. When MTA sets completely in a dry condition, it has a thick microstructure that contains residual partially hydrated grains. ^28^ The surface roughness of the materials might have decreased due to the loss of hydration over time. In wet conditions, the surface roughness of ProRoot MTA decreased on the 1^st^ day, which may result from washout of MTA due to its slow setting properties. ^29^ However, a trend of increased surface roughness of both materials in wet conditions was found. When the material completed setting in 3 days, the excess water might have prolonged the dissolution of the biomaterials, leaving a more porous outer surface on the remaining material. The increased roughness in wet conditions might also be due to the material properties. This can be related to the accumulation of globular deposits on the surface of the materials after storage in physiological solution. ^20^ Regarding the effects of acidic condition, Smith, et al. ^18^ (2007) examined the surface characteristics of ProRoot MTA after exposure to EDTA (pH 7.4) and Biopure MTAD (pH 2) and reported increased surface roughness and higher calcium release in MTAD-treated specimens. However, in this study, the acidic pH did not affect the surface roughness of the materials. In blood conditions, ProRoot MTA and Biodentine showed high roughness values up to 3 days but a significant decrease after 28 days. The high surface roughness of the materials might be related to the accumulation of blood proteins on the surfaces of the materials. ^30^

The initial roughness values of ProRoot MTA and Biodentine were similar in this study. Similarly, a previous study measured the surface roughness using an atomic force microscopy and reported similar values for ProRoot MTA and Biodentine. ^15^ However, the exposure of materials to different environmental conditions can result in different morphological changes on the surface. ^31^ A previous study reported that both materials formed white precipitates consisting of hydroxyapatite crystals on their surfaces after exposure to PBS. ^32^ The formation of a white precipitate in a different quantity on the surface of the materials may explain the higher surface roughness of Biodentine compared with ProRoot MTA on days 1 and 3 in wet conditions. ^33^

In dry conditions, ProRoot MTA showed no dimensional change while Biodentine showed 0.47% shrinkage after 28 days. In agreement with our results, Camilleri, et al. ^20^ (2014) reported structural changes, crack formation and shrinkage of Biodentine when stored in dry conditions. In wet conditions, ProRoot MTA expanded by 0.41% and 0.17% on days 3 and 28, while Biodentine expanded by 0.85% and 1.44%. The greater expansion in Biodentine may stem from the higher accumulation of hydroxyapatite on the surface of the material due to its higher ability to release calcium in synthetic tissue fluids. ^34^ In the acidic environment, the surface level of both materials was constant during all time periods. The different effects of acidic and neutral pH levels on the properties of the materials might be related to inhibition of the setting reaction, which may lead to the fast dissolution of the materials in an acidic environment. ^35^ For this reason, the solubility of the material may impair the dimensional expansion by preventing the accumulation of hydroxyapatite on the material surface. In blood conditions, both materials decreased in volume. The crystalline phases of the materials during hydration are also important to understand, as well as the cement composition and changes that occur during the setting process. By hydration, both materials showed poorly crystallized amorphous calcium silicate hydrate gel that contains globular particles and micro channels. ^10^ This calcium silicate hydrate gel and the formation of calcium hydroxide provide the setting and contribute to the strength of these cements by the precipitation of hydroxyapatite crystals. ^36^ In a previous study, the exposure to the blood conditions for four days did not induce hydroxyapatite crystal formation on the surface of white ProRoot MTA. ^37^ This might be related to the prohibition of the hydration process by blood contamination, which prevents the formation of this crystal structure and leads to unset materials. ^38^ Additionally, exposure to blood reduced the formation of calcium hydroxide by ProRoot MTA. ^39^ For this reason, the superficial reduction of the materials by blood contamination can be related to the inhibition of the hydration and hydroxyapatite formation. Similarly, Grench, Mallia and Camilleri ^33^ (2013) reported higher wash-out tendency of Biodentine, with the loss of material upon contact with blood and other fluids. Unset materials might have been washed out after exposure to the blood conditions in this study.

Overall, the roughness of both materials decreased after 28 days in dry conditions, which may be related to the loss of hydration over time. Both materials increased surface roughness and expanded in wet conditions through time, which may improve the clinical performance of the materials when used in humid environments. In blood condition, both materials presented decreased surface roughness and shrinkage through time and this can be a disadvantage for apical surgery or revascularization treatments, as it can disrupt adaptation and lead to microleakage. In acidic conditions, the materials showed no significant changes in terms of surface roughness and dimensional change, which may be related to the increased solubility and reduced bioactivity properties of the materials. Further studies are required to clarify the clinical importance of these changes.

## Conclusions

The environmental conditions tested had different effects on the surface of ProRoot MTA and Biodentine. The storage in dry, wet, and blood conditions had a time-dependent effect on the surface roughness and vertical dimensional changes of the materials. However, the roughness and the surface level of the materials were not affected by the storage in acidic conditions.
